# Fabrication of a Monolithic Lab-on-a-Chip Platform with Integrated Hydrogel Waveguides for Chemical Sensing

**DOI:** 10.3390/s19194333

**Published:** 2019-10-08

**Authors:** Maria Leilani Torres-Mapa, Manmeet Singh, Olga Simon, Jose Louise Mapa, Manan Machida, Axel Günther, Bernhard Roth, Dag Heinemann, Mitsuhiro Terakawa, Alexander Heisterkamp

**Affiliations:** 1 Institute of Quantum Optics, Gottfried Wilhelm Leibniz University Hannover, 30167 Hannover, Germanyheisterkamp@iqo.uni-hannover.de (A.H.); 2 Lower Saxony Centre for Biomedical Engineering, Implant Research and Development (NIFE), 30625 Hannover, Germany; 3 Industrial and Biomedical Optics Department, Laser Zentrum Hannover e.V., 30419 Hannover, Germany; 4 School of Integrated Design Engineering, Keio University, Yokohama 223-8522, Japanterakawa@elec.keio.ac.jp (M.T.); 5 Hannover Centre for Optical Technologies, Gottfried Wilhelm Leibniz University Hannover, 30167 Hannover, Germanybernhard.roth@hot.uni-hannover.de (B.R.); 6 Cluster of Excellence PhoenixD (Photonics, Optics and Engineering-Innovation Across Disciplines), 30167 Hannover, Germany

**Keywords:** waveguide, microfluidics, fluorescence, hydrogels, 3D printing

## Abstract

Hydrogel waveguides have found increased use for variety of applications where biocompatibility and flexibility are important. In this work, we demonstrate the use of polyethylene glycol diacrylate (PEGDA) waveguides to realize a monolithic lab-on-a-chip device. We performed a comprehensive study on the swelling and optical properties for different chain lengths and concentrations in order to realize an integrated biocompatible waveguide in a microfluidic device for chemical sensing. Waveguiding properties of PEGDA hydrogel were used to guide excitation light into a microfluidic channel to measure the fluorescence emission profile of rhodamine 6G as well as collect the fluorescence signal from the same device. Overall, this work shows the potential of hydrogel waveguides to facilitate delivery and collection of optical signals for potential use in wearable and implantable lab-on-a-chip devices.

## 1. Introduction

The past few years have seen rapid growth and interest in soft and pliant materials due to their applications in various fields such as robotics [1,2], health monitoring [3] and drug delivery [4]. Wearable technologies are foreseen as devices that will enable personalised and continuous recording of physiological signals. Such devices include a sensing element for analysis combined with a microfluidic platform in order to facilitate liquid collection in micro-liter volumes [5]. The potential of such systems for sensing biomolecules, metabolites and electrolytes in bodily fluids have been demonstrated in several studies [6,7,8]. For example, wearable microfluidic devices have been used to perform continuous monitoring of lactase and glucose levels in sweat during exercise [7]. Contact lenses are being considered as a non-invasive quantitative device to measure glucose in human tears. This spurs studies on developing techniques to engrave microfluidic channels in contact lenses to facilitate tear collection [9]. Owing to their biocompatibility and tunability, hydrogels and elastomers are currently being explored as candidate materials for stretchable functional devices [10,11,12].

Optical detection methods such as laser induced fluorescence for sensing offers an ideal approach for such wearable devices due to their sensitivity, fast response time and reliability. Integration of flexible optical elements into the wearable microfluidic device allows a more compact footprint and reduces the need for bulky optics. Commonly, optical fibers are used which are often made of pure silica as cladding surrounded with a core of silicon-doped material. Although, they can robustly deliver light up to several kilometers with minimal loss (−70 dB/km), they can be potentially hazardous and cause injury when they break. Recently developed polymer fibers made of synthetic chemicals such as poly(methyl methacrylate) are also being explored as waveguides for biomedical sensing applications [13,14]. Durable and long-lasting, they are potentially useful for wearable technologies and in vivo applications. Aside from material properties, such as biocompatibility, an easy fabrication method which does not require expensive and large fiber drawing facilities would also be ideal.

An attractive waveguide to integrate in flexible wearable platforms are hydrogels which are characterized as three-dimensional polymer networks formed by crosslinked hydrophilic polymer chains and are able to absorb water. In this work, we explore the use of polyethylene glycol diacrylate (PEGDA) hydrogel as a waveguide to demonstrate light guiding and optical signal collection from a solution in a microfluidic channel. PEG-based hydrogels can be synthesized with a variety of cross-linking densities and are being explored for various applications particularly in tissue engineering and drug release. Compared to other types of waveguides that have been integrated in microfluidic devices, fabrication of hydrogel waveguide is very simple and have high fidelity to the original design. Although, a relatively new material as an optical waveguide, PEGDA has been applied to different scenarios such as: (1) measurement of oxygen levels by detecting changes in optical signals in vivo [15], (2) wound closure via photochemical absorption of Rose Bengal [16] and (3) initiate insulin release on genetically engineered light-sensitive cells [17]. On their own, hydrogels can also be used to guide light in ambient air. The rationale behind such hydrogel waveguide design is to obtain direct signals from the environment, which changes the optical and volumetric properties of the hydrogel. This can be both an advantage and a disadvantage. On one hand, the increased sensitivity will enable sensing of minute changes in the tissue environment. For example, PEG bonded to polyacrylamide hydrogels incorporated with a glucose responsive monomer can be used for in vivo glucose monitoring [18,19]. However, the sensitive nature of hydrogels also leads to unstable long-term optical measurements especially in a dynamic environment.

Polydimethylsiloxane (PDMS) is a well-known material especially in microfluidics and has been shown to be both biocompatible and oxygen permeable [20]. A hybrid waveguide made of hydrogel as the light guiding core and PDMS as its low index cladding, can provide a robust waveguide for light guiding and signal collection. Hence, this work explores the integration of hydrogel waveguides in a microfluidic device made of elastomeric material such as PDMS towards a soft and biocompatible lab-on-a-chip platform for stable long-term chemical sensing in a microfluidic format. We investigated the swelling and optical properties of PEGDA hydrogels with different molecular weights and concentrations. Using simple and affordable micro-molding techniques supported by 3D printing technology, which does not require clean-room facilities, waveguides and channels can be easily implemented in a microfluidic chip for lab-on-a-chip application. Rapid prototyping of the molds for the microfluidic chip and waveguides was performed with a high-resolution 3D printer. This enabled us to produce molds reproducibly with acceptable surface roughness and precise channel dimensions. We further demonstrate the use of such integrated systems for chemical sensing using laser induced fluorescence.

## 2. Materials and Methods

All chemicals were purchased from Sigma-Aldrich (St. Louise, MO, USA) and used as is, unless specified otherwise. PEGDA with molecular weight: 250, 700, 2000, 4000 (Polysciences, Inc., Warrington, PA, USA), 6000 Da were used in the following experiments.

### 2.1. Fabrication of Waveguides

Molds with the positive relief of the channels and waveguides were designed using CAD software (Solidworks, Dassault Systèmes, Vélizy-Villacoublay, France). The design was saved in stereolithography file (STL) format and printed using a UV curable material (VeroGray RGD850, Stratasys, Rehovot, Israel) with a 3D printer (Eden 260 V Stratasys, Rehovot, Israel). After printing, the support material (Support SU705, Stratasys, Rehovot, Israel) was removed using a pressurized waterjet. Sylgard 184 elastomer (Dow Corning, Midland, MI, USA) was mixed thoroughly in a 10:1 (base:curing agent) ratio and then poured into 3D printed molds. The solution was degassed for at least 35 min and then heat-cured at 125 ${}^{\circ }$C in a preheated oven for 55 min. PDMS substrate was completely cooled before peeling from the molds. The cured PDMS were corona-treated (BD-20ACV, Electro-Technic Products, Chicago, IL, USA) to bond the structures together. Corona treatment relies on high voltage discharge in the air to improve wettability and adhesion of PDMS. Prior to filling the channels with the precursor hydrogel solution, the structures were placed in a vacuum chamber to remove air bubbles. PEGDA 700, at 90 wt% mixed with a crosslinker 1 wt% 2-Hydroxy-4-(2’-hydroxyethoxy)-2-methylpropiophenone (Irgacure 2959) in water were injected into the waveguide channels and polymerized via free-radical polymerization under UV lamp (Vilber Lourmat, Collégien, France, *P* = 3 W, λ = 365 nm) for 7–8 min. Waveguides were directly used after fabrication to prevent dehydration of PEGDA core. Most PEGDA are soluble in water except for PEGDA 250. Instead, PEGDA 250 was mixed with 1 wt% irgacure dissolved in 70 v% ethanol. Straight waveguides were also fabricated by inserting needles with different diameters in the PDMS on rectangular molds. After curing the PDMS, the needle was carefully removed forming a channel where the precursor solution can be injected. Channels on the PDMS were treated with 10% benzophenone dissolved in 99.99% ethanol for at least 10 min [12].

### 2.2. Optical Characterization

Fabricated hydrogels for transparency measurements were prepared using chamber slides (IBIDI, Martinsried, Germany) as molds with dimensions of 9.4 × 10.7 × 6.8 mm. 250 μL volume of the precursor solution was used. Gels have the same dimension as the molds directly after fabrication with a thickness of ≈2.1 mm. Indices of refraction for the materials were determined with an Abbe refractometer (AR4, A. Kruess Optronic GmbH, Hamburg, Germany) at room temperature. Loss measurements were performed using cut-back method for different waveguide diameters. A 532-nm microchip laser (HLX-G-F020, Horus Laser, Limoges, France) operated at 1 ns pulse width with repetition rate of 22.5 kHz was focused to a spot size of about 5 μm at the facet of the waveguides. Measurements of power output was performed using a power meter. Spectral transmission curves of the gels were obtained by curing PEGDA mixed with 1 wt% irgacure 2959. A total volume of 600 μL of the precursor solution was pipetted in a cuvette, cured for 3 min for 40, 60, 80, 90 wt% and 1 h for 20 wt% under a UV lamp (Vilber Lourmat, Collégien, France, *P* = 6 W, λ = 365 nm) and measured directly afterwards with a spectrometer (UV-3600 Plus, Shimadzu, Kyoto, Japan) with a path length of 10 mm.

### 2.3. Swelling Ratio, Water Content and Water-Induced Volume Swelling

The hydrogels were fabricated, weighed, swollen in Milli-Q water (MilliporeSigma, Burlington, MA, USA) and then freeze-dried. The gels were fabricated with the same molds used in the transparency experiments and then weighed at different time points of immersion in water. The swelling ratio, *Q*_m_ was calculated using the equation:

(1) $$Q_{m}=\frac{m_{\mathrm{sw}}-m_{d}}{m_{d}}\times 100\%$$

wherein *m*_sw_ is the gel’s weight after swelling at a certain time and *m*_d_ is the gel’s freeze-dried weight. The water content (W_c_) was obtained from the percentile ratio of *m*_sw_ and *m*_d_. Water-induced volume swelling ratio (*Q*_w_) was obtained by using the relation [21],

(2) $$Q_{w}=\frac{\nu_{2,r}}{\nu_{2,s}}$$

where ν_2,r_ is the volume fraction of the hydrogel directly after polymerization (relaxed state of the gel) and ν_2,s_ is the volume fraction of the hydrogel after swelling in water. These volume fractions can be obtained using the following relations:

(3) $$\nu_{2,s}={\left[1+\frac{\left(\frac{m_{\mathrm{sw}}}{m_{d}}-1\right)\rho_{h}}{\rho_{H_{2}O}}\right]}^{-1}\mathrm{and}\;\nu_{2,r}={\left[1+\frac{\left(\frac{m_{p}}{m_{d}}-1\right)\rho_{h}}{\rho_{s}}\right]}^{-1}$$

where ρ_h_ is the density of the hydrogel. For PEGDA, the density is ρ_p_ = 1.12 g/cm^3^. ρ_s_ is the density of the solvent used in hydrogel polymerization and m_p_ is the mass of the hydrogel directly after polymerization. Ethanol (ρ_EtOH_: 0.79 g/cm^3^) was used to fabricate PEGDA 250 hydrogels while water was used for PEGDA 700 and 6000 (ρ_H_2_O_: 1.00 g/cm^3^).

### 2.4. Chemical Sensing

Measurements were performed with the same laser used for optical characterization. The beam was directed to a half-wave plate paired with a polarizing beam splitter in order to control the power of the incident laser beam. The beam was magnified using a pair of plano-convex lenses (*f* = 35 mm and *f* = 125 mm), directed by a couple of mirrors and focused to a spot size of ∼5 μm using a lens (*f* = 75 mm) to the input end facet of PEGDA waveguide. The entire chip was mounted securely on a 3-axis micrometer positioning stage (Elliot Scientific, Harpenden, UK) in order to align the input end facet of the waveguide to the focused laser beam. Another pair of lenses (both *f* = 100 mm) at the output distal end of the second PEGDA waveguide was used to collect the optical signal from the chip. The output signal from the chip was filtered by a band pass filter (Brightline fluorescence 593/40, Thorlabs, Newton, NJ, USA) and the detected fluorescence was measured by a fiber-based spectrometer (CCS200/M, Thorlabs, Newton, NJ, USA) using a fixed integration time of 100 ms.

Rhodamine 6G was dissolved in Milli-Q water (MilliporeSigma, Burlington, MA, USA) with a stock concentration of 1 mg/mL. The stock solution was further diluted in Milli-Q water to obtain different concentrations.

## 3. Results

In order to determine which type of PEGDA and concentration is optimal as optical waveguides to integrate in a microfluidic chip, we performed a general assessment of the relevant properties of different variety of polymerized hydrogels. Since molecular weight (MW) determines many properties of the hydrogels such as cross-linking density, mesh size and diffusion, PEGDA with different MW and mass per volume concentration were characterized for swelling ratio and water content. We also assessed the water-induced volume changes and the optical properties of the fabricated PEGDA hydrogels. The physical parameters of the hydrogels in a water environment could also affect its waveguiding properties.

**Figure 1:**
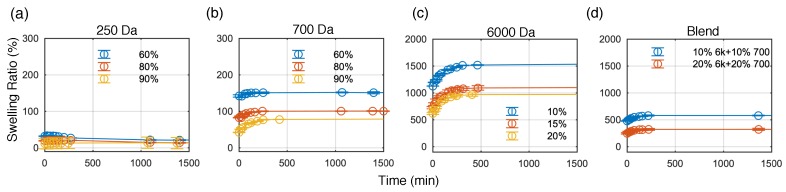
Swelling ratio as a function of time for (**a**) 250 Da; (**b**) 700 Da; (**c**) 6000 Da and (**d**) blend of 700 Da and 6000 Da. At *t* = 0, swelling ratio is determined by the weight of the hydrogel directly after fabrication with respect to the dried weight of the hydrogel.

Hydrogels in general swell and shrink depending on their environment due to their mesh-like structure. Network mesh size increases with increasing MW and correspondingly will facilitate higher intake of water. This was also observed in our experiments. Figure 1a–d shows the swelling ratio at different time points for different PEGDA. Except for PEGDA 250, most of the fabricated PEGDA hydrogels exhibit a fast increase in swelling ratio which reach equilibrium after being immersed in water for several hours. The measurements were performed up to 15,000 min. No significant changes in swelling ratio was observed beyond 1500 min.

**Figure 2:**
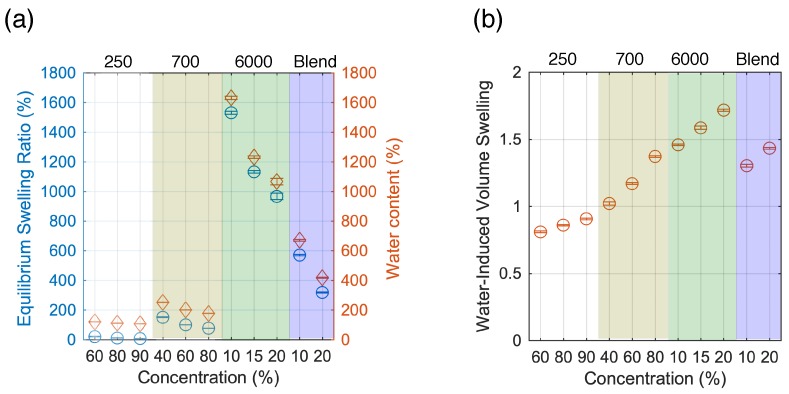
(**a**) Equilibrium swelling ratio and water content for different molecular weight and concentration; (**b**) Calculated water-induced volume swelling of polyethylene glycol diacrylate (PEGDA) for different concentration and molecular weight.

The equilibrium swelling ratio and calculated water content are further summarized in Figure 2a. Higher MW, that is, PEGDA 6000 (*Q*_m_ ≈ 1600%) results to higher swelling ratio and water content compared to PEGDA 250 and 700 (*Q*_m_≈ 200%). Although the swelling ratio depends on both the MW and concentration, it is more strongly affected by MW. Increasing the concentration of PEGDA for the same MW, which leads to a tighter network, results to a decrease in both swelling ratio and water content. On the other hand, calculated water-induced volume swelling ratio increases with increasing concentration as well as MW (Figure 2b). Whereas the swelling ratio and water content provide information about the water absorbed during the swelling of the hydrogel, the water-induced volume swelling indicates the volume change of the hydrogels. Among the PEGDA tested for our experiment, PEGDA 250 and PEGDA 700 did not exhibit drastic changes in volume after immersing in water as compared to PEGDA 6000. However, PEGDA 250 yielded very stiff and brittle hydrogels and exhibits material loss during the swelling experiments. The mixture of PEGDA 6000 and 700 showed interesting swelling properties with reduced swelling ratio and water content as well as water induced-volume swelling compared to single network PEGDA 6000. Weight of the fabricated hydrogels reaches equilibrium approximately 250 min after placing in water as shown in Figure 1. It is thus recommended to equilibrate the gels for at least 4h before using hydrogels in water environment. In air, gel shrinks and decreases in weight due to loss of water (data not shown).

**Figure 3:**
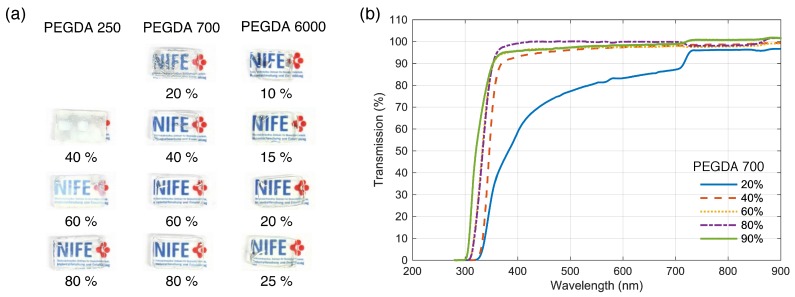
(**a**) Transparency of the hydrogels for different molecular weight and concentration; (**b**) Optical transmission of PEGDA 700 hydrogels for different concentrations.

Optical properties of the biocompatible optics will largely depend on the transmission properties and the refractive index. By varying the concentration and the molecular weight of the PEGDA, the transparency as well as the transmission properties of the hydrogel changes [17]. We confirmed this in our experiments. For lower MW, the concentration at which hydrogels becomes transparent is higher compared to higher MW PEGDA. For example, PEGDA 250 is opaque for concentrations less than or equal to 80% but becomes transparent with higher concentrations (Figure 3a). For PEGDA 700, transparency is achieved at MWs greater than 40% whereas for PEGDA 6000, hydrogels are transparent even at low concentration such as 10%. PEGDA 2000 and 4000 were also transparent at concentrations ≥15%. Optical transmission is higher than 90% for most concentration of PEGDA 700 for wavelengths longer than 400 nm (Figure 3b).

**Figure 4:**
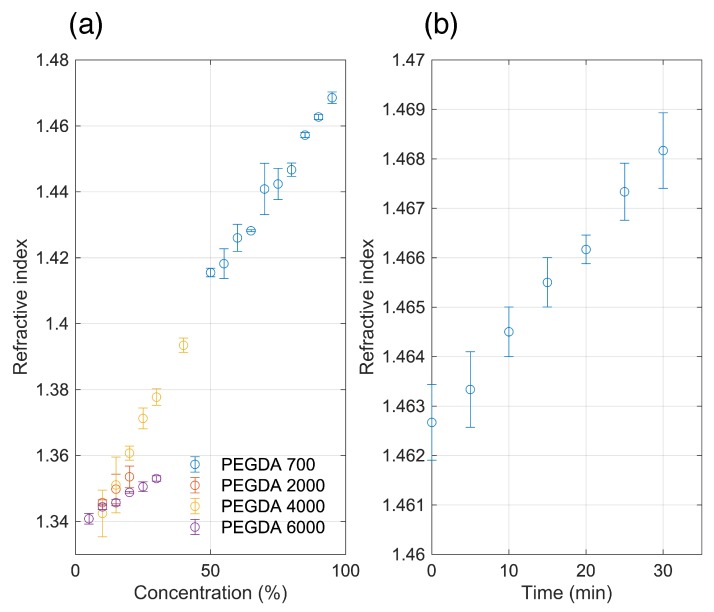
(**a**) Refractive indices of PEGDA hydrogels at different molecular weights and concentrations; (**b**) Refractive index of PEGDA 700, 90% left in air over time. Each data point is an average measurement of n = 3 hydrogels.

The refractive index determines the numerical aperture (NA) of the waveguides fabricated. NA of the waveguide affects the amount of light collected especially important for sensing applications. The NA of the waveguide is given by the following equation,

(4) $$NA=\sqrt{{n_{core}}^{2}-{n_{cladding}}^{2}}$$

where *n_core_* and *n_cladding_* is the refractive index of the core and cladding, respectively. Hence, the refractive indices of the hydrogels were also characterized for different concentrations and MWs. The refractive indices linearly increase with increasing concentrations. For PEGDA 700, refractive index measurements that can be performed with the Abbe refractometer occur at concentrations 45% and above, such that hydrogels are polymerized and transparent. For PEGDA 2000, 4000 and 6000, the refractive indices can be measured at much lower concentrations than PEGDA 700. However, at these concentrations the refractive indices are closer to the refractive index of water. For PEGDA 700, 95% the refractive index can reach up to 1.469 ± 0.002 (Figure 4a).

We also measured the refractive index change over time with PEGDA 700, 90% hydrogel exposed to air and observed a small but measurable linear increase in refractive index due to water loss in the material (Figure 4b). From 1.463 ± 0.001 directly after fabrication, PEGDA 700, 90% refractive index reaches up to 1.468 ± 0.001 after 30 min in air. The results were an average measurements of three separately fabricated hydrogels.

Altogether, PEGDA is a suitable material for creating flexible optical waveguide due to its high transmission, predictable swelling behavior and tunable refractive index. By choosing the appropriate MW and concentration, one can carefully tune the optical and mechanical properties to create the desired optics. Among the different molecular weight and concentration tested, PEGDA 700, 90% has one of the highest indices of refraction with stable transmission output and therefore is a suitable core for the waveguide. We tested waveguiding with this core material combined with a lower concentration PEGDA 700, 40% as the cladding. (Figure S1a). Waveguiding can be clearly and reproducibly observed with light guided in the core (radius = 400 μm) of the fabricated 3 cm waveguide. This effect can be seen even 10 weeks after fabrication (Figure S1b). However, waveguides fabricated were extremely fragile, prone to misalignment and very sensitive to environment. We also observed that light transmitted and the structure itself can be very unstable after several minutes especially with thin waveguides which we attribute to material dehydration.

PDMS has a refractive index of 1.412 ± 0.004 (*n* = 3 samples) at our fabrication condition and is a suitable cladding for most PEGDA 700 concentration measured. Higher MW PEGDA at concentrations we tested are not compatible as a core material due to lower refractive indices compared to PDMS. As a cladding and flexible substrate for wearable platforms, PDMS provides a robust material that prevents the dehydration of the waveguide and maintains its shape and functionality. An elastomer such as PDMS also reduces the shrinkage of hydrogels [12]. This translates to a more stable optical transmission over time for the cladded waveguide. PDMS is also transparent and allows for UV polymerization of the precursor hydrogel solution. Fabrication of hydrogel waveguides cladded with PDMS is a straightforward procedure using already established soft lithography techniques. Waveguides were also flexible and can be bent albeit with increasing losses with increasing bending angle as shown in Figure S2.

**Figure 5:**
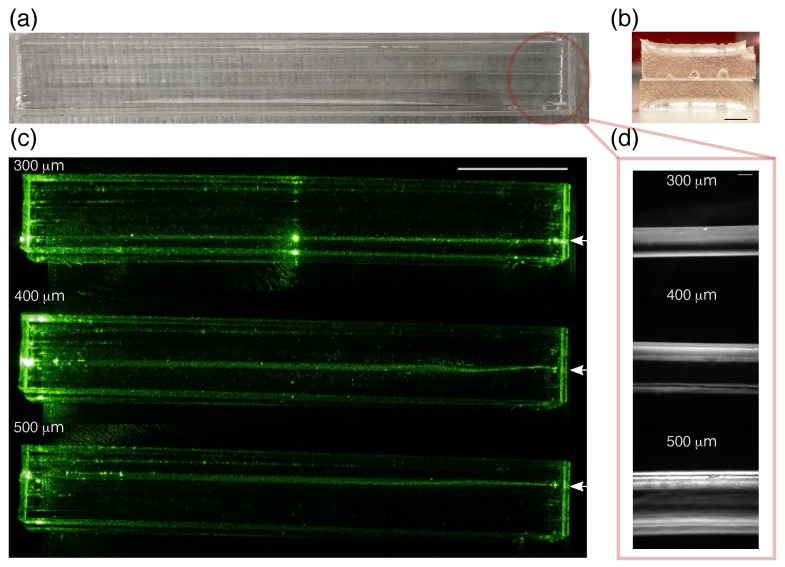
(**a**) Photograph of the PEGDA waveguides embedded in polydimethylsiloxane (PDMS). Three PEGDA 700, 90% waveguides with different radii were embedded in a single PDMS block for waveguiding tests and cut-back measurements; (**b**) Photo of the cross-section of the waveguide. Scale bar is 20 mm; (**c**) Representative photos of a fabricated 10 cm straight waveguide in a PDMS cladding with the 532 nm laser guided along the PEGDA 700, 90% core. The focused laser is incident to the waveguide marked by the arrow. Scale bar is 25 mm; (**d**) Microscope images of the fibers fabricated by filling the PDMS channels with PEGDA 700, 90% with different radii. Scale bar is 300 μm.

In order to assess the loss through the waveguides, cores with different radii were fabricated surrounded with PDMS cladding. Waveguides in this case are termed step-index and support thousands of modes. Figure 5a,b show representative photos of the straight waveguides cladded with PDMS showing the half-spherical shape of the PEGDA cores using our fabrication procedure. Light can be guided along the entire 10 cm waveguide length as shown in Figure 5c. Strong intense scattering at one point in the 300 μm waveguide could be due to a crack formed at the PEGDA core. Losses were measured using cutback technique for the waveguides fabricated. The measurements yielded propagation losses of 1.00 ± 0.04 dB/cm, 0.95 ± 0.01 dB/cm and 0.85 ± 0.06 dB/cm for 300 μm, 400 μm, 500 μm core radii respectively. Measured power for the three waveguide diameters were stable over the course of 30 min (Figure S3a). Figure 5d shows microscopy images of the PEGDA cores. Smooth structures can be seen with dimensions reproducible for every fabrication. Straight waveguides were also fabricated using commercially available needles to account for the surface roughness of the PDMS after demolding. Losses were observed to be less for waveguides fabricated using commercially available needles (≈0.81 dB/cm for radius = 360 μm). Potential sources of losses can be due to presence of microcracks in the PEGDA 700 waveguide core, scattering due to the roughness of the PEGDA waveguide and the PDMS channels, as well as the slight curvature of the fabricated waveguides.

**Figure 6:**
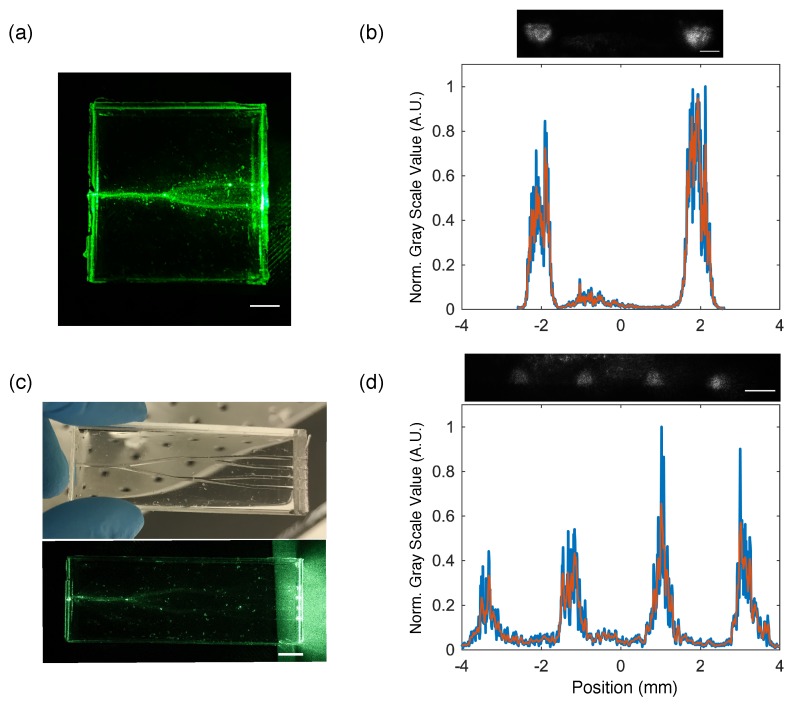
(**a**) A photo of a waveguide splitter with PEGDA 700, 90% waveguide and radius of 300 μm Scale bar is 0.5 cm; (**b**) Plot is the line profile of the beam output showing the light distribution at the two distal ends of the y-splitter. Top image shows the beam output from the y-splitter. Scale bar is 0.5 cm; (**c**) (**Top**) A photo of a fabricated 1 × 4 waveguide splitter with waveguide radius of 300 μm; (**Bottom**) A photo of the 1 × 4 waveguide splitter showing four intense light output at the distal ends. Scale bar is 1.0 cm; (**d**) Top image shows the four points as imaged in the CCD camera. Scale bar is 0.5 cm. Plot shown is the line profile of the 4 spots from the 1 × 4 splitter. For plots in (**b**,**d**) blue curve is the normalized gray scale value and red curve is a Savitzky-Golay fit with order 2 and frame length 17.

With the 3D printed molds, the fabrication process allows us to easily design waveguides for variety of applications. For example, we designed waveguide splitters with a core material PEGDA 700, 90% and cladding using PDMS. Figure 6a shows an example of the fabricated waveguide splitter and the corresponding output beam profile from the splitters (Figure 6b). Each arm has a length of 2 cm and a main arm with a length of 1 cm. The y-splitter has an opening angle of 20${}^{\circ }$. Two beams can be observed at the output end with very minimal loss at the splitter junction. However, inhomogeneity in the beam profile was observed which can be attributed to imperfect fabrication due to formed cracks at the PEGDA waveguide. Speckled intensity pattern is also evident due to the multimode nature of the waveguide. A 1 × 4 waveguide splitter can also be fabricated as shown Figure 6c,d. The main splitter has an opening angle of 20${}^{\circ }$ and the smaller arms have an opening angle of 10${}^{\circ }$. Four intense beams can also be observed showing the combination of PEGDA and PDMS can be used to efficiently split light into multiple regions on a chip if necessary. In order to provide a measure of the optical field of the light guided by the fabricated waveguides, the mode field diameter which is defined as the radial position wherein the intensity is reduced to 1/e${}^{2}$ or ≈ 0.135 of its maximum value was calculated from the intensity profile of the splitters. The estimated mode field diameter is 670 ± 40 μm for the 1 × 2 splitter and 825 ± 112 μm for 1 × 4 splitter.

**Figure 7:**
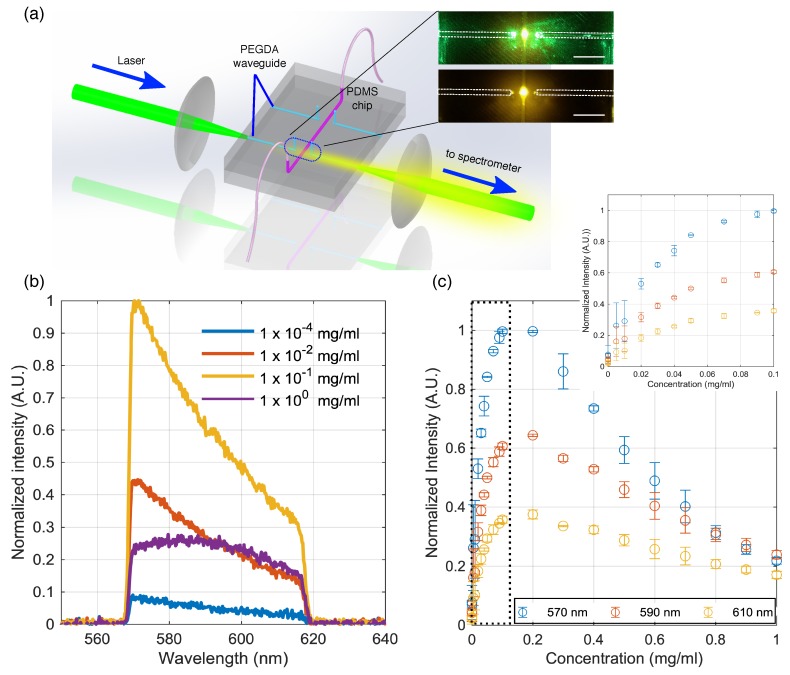
(**a**) Schematic diagram of the optical setup and a photo of the microfluidic chip with an integrated hydrogel waveguide. Laser is focused at the entrance of a PEGDA waveguide and a second PEGDA waveguide was used to collect the laser-induced fluorescence signal generated at the microfluidic channel. Top image shows a photo of the chip with the 532 nm laser guided to the microfluidic channel. Bottom shows a photo taken with a bandpass filter to allow only the fluorescence emission to be captured by the camera. Dotted lines indicate the location of the PEGDA waveguides. Scale bar is 0.5 cm; (**b**) Representative spectral profiles of laser induced fluorescence of rhodamine for different concentrations; (**c**) Normalized intensity of the rhodamine emission signal as a function of concentration for 570, 590 and 610 nm. Inset graph shows the curve between 0 to 0.1 mg/mL. Data shown is an average measurements from three different microfluidic chips.

Based on our measurements, we confirmed that the fabrication protocol produces waveguides with sufficient optical performance, characterized with multimode output with higher losses compared to waveguides molded with commercially available needles. Based on its swelling and optical properties, PEGDA 700, 90% was chosen to integrate in a microfluidic chip (Figure 7a). Using Equation (4) to calculate the NA for a step-index waveguide, wherein n_core_ and n_cladding_ is the refractive index of PEGDA 700, 90% and PDMS respectively, we calculated a NA of 0.39 for our fabricated waveguides.

A microfluidic channel with a diameter of 750 μm was constructed between a pair of PEGDA waveguides (*r* = 300 μm) each with length of 1.2 cm. Between the PEGDA waveguide and the solution in the channel is a 2 mm thick PDMS wall. One of the waveguides was used to deliver the laser to the channel and excite rhodamine 6G solution in the microfluidic channel while the second PEGDA waveguide collects the fluorescence signal as shown in Figure 7a. A band pass filter cuts-off the excitation laser as well as any contributory emission light below 570 nm at the detection arm. Figure 7b shows representative spectral profiles of some of the rhodamine concentration tested in the experiment. Rhodamine exhibits a broad emission spectral profile with peak at 570 nm which tails off at 620 nm. The fluorescence intensity as a function of rhodamine concentration for spectral wavelengths: 570, 590 and 610 nm are shown in Figure 7c. Measurements were performed using 3 different microfluidic chips each with freshly fabricated PEGDA waveguides. At concentrations below 1 ×$10^{-1}$ mg/mL, an increase in intensity signal is observed with increasing concentration. Beyond this concentration, the intensity decays for all three wavelengths.

A red shift in peak emission wavelength was also observed for higher concentrations. Previous studies have shown that the presence of aggregates forms dimers which changes the electronic structure and the emission properties of rhodamine 6G [22,23]. Therefore, the signal intensity at concentrations greater than 1 ×$10^{-1}$ mg/mL is reduced and the peak emission shifts to higher wavelengths which is consistent with earlier spectroscopy measurements on rhodamine 6G [23]. The lowest rhodamine concentration used for the measurements was 1 ×$10^{-5}$ mg/mL. The limit of detection (LOD) which is defined as the lowest concentration which can be measured by the system, was estimated using the relation, $LOD=\frac{3\sigma }{M}$ where σ is the standard deviation of the measurements of a blank sample, in our case, water was used, determined from 10 individual measurements [24]. *M* is the slope of the linear fit for concentrations 7 ×$10^{-2}$ mg/mL and below where a linear relationship between the emission curve and rhodamine concentration was observed. Estimated LODs were 9.0 ×$10^{-3}$, 1.53 ×$10^{-2}$ and 2.59 ×$10^{-2}$ mg/mL for 570, 590 and 610 nm respectively. The LOD can be reduced further with a more optimized detection optics by using a higher numerical aperture lens to collect emission from the chip and increasing the integration time of the spectrometer. We also monitored the stability of the measurements under room conditions by measuring the spectral output every 5 min for 25 min and did not observed significant changes on the emission intensity measured from the microfluidic chip (Figure S3b).

## 4. Discussion

This work describes the integration of flexible hydrogel waveguides in a microfluidic device as a lab-on-a-chip platform for laser induced fluorescence. Elastomers and hydrogels are currently being proposed as building blocks for wearable technologies in order to perform body fluid measurements as well as initiate targeted drug release. PEGDA hydrogels exhibit tunable optical and mechanical properties with multiple possibilities for functionalization. Multimode polymer fibers [25] and femtosecond laser inscribed waveguides in glass [26] have been also previously integrated in a microfluidic format and used for waveguiding in lab-on-a-chip applications. Similarly, extensive work has been done in the field of optofluidics, which uses fluids with controllable refractive index to guide light [27]. Hydrogel waveguides integrated in the PDMS are self-aligned and fabrication is relatively simple via micro-molding technique which enables easy merging to a lab-on-a chip device.

PEGDA waveguides embedded in PDMS exhibit high transmission, minimal absorption at visible wavelengths and low propagation losses (less than 1.1 dB/cm). Similar losses of 1 dB/cm in polymer fibers have been measured [28]. Step index hydrogel waveguides demonstrated in literature such as PEGDA 700 and polyacrylamide acting as core of waveguides with alginate cladding reported losses of 3.4 dB/cm [15] and 0.45 dB/cm [29], respectively. PDMS pre-polymer was also used in microfluidic chips with measured losses of 1.8 dB/cm for 532 nm [30]. The losses we have stated here are comparable and, in some cases, even lower than other hydrogel waveguides available in literature. Therefore, PEGDA 700, 90% and PDMS are compatible flexible materials for waveguiding. In comparison to other flexible substrates reported in literature such as PDMS waveguides incorporated in PDMS chips, the use of hydrogel waveguide exhibits a wider range of refractive index and swelling properties. The appropriate concentration and molecular weight PEGDA offer control on the swelling or volumetric change.

For use in a possible scenario where high degree of bending and strain could occur, PEGDA 700 at 90% is comparably stiffer to high molecular weight PEGDA. It is thus possible that microcracks on PEGDA may form during bending could lead to scattering and loss. Increase toughness can be achieved by mixing long chain with short-chain length hydrogels or in interpenetrating form and may offer enhanced tensile strength and flexibility [31,32]. For applications related to wearable technologies, depending on the degree of flexibility and stretching needed –investigating both the mechanical and light-guiding properties during stretching and bending is necessary.

In this work, we have explored the use of PEGDA hydrogel waveguides in microfluidic devices. Since waveguide splitters and other geometrical forms are possible, on-chip optical sensors such as Mach-Zender interferometers can also be fabricated. Additionally, fabrication of the master mold is performed via high resolution 3D printing, which facilitates parallel and rapid prototyping, thus, multiple optical elements made of hydrogels can be implemented in the device such as lenses and diffusers. Lenses could be fabricated directly on-chip to improve collection efficiency of fluorescence emission. Silver gratings [33] as well as electronically conductive materials [34] have been inscribed in PEGDA hydrogels using femtosecond lasers and shows the potential of combining these materials to functional metallic nanomaterials for plasmonic, diffractive or electronic sensing. Integrating such metallic structures to the device could enhance the intensity of the fluorescence emission via plasmonic interaction [35], thus, could further improve the signal to noise ratio, limit of detection and sensitivity, which are critical properties of wearable microfluidic devices.

## Supplementary Materials

The following are available online at https://www.mdpi.com/1424-8220/19/19/4333/s1, Table S1: Design dimension of the molds and the corresponding fabricated PEGDA waveguide radii (*n* = 2), Figure S1: (**a**) Waveguiding using PEGDA 700, 90 % (*n* = 1.462) as core with a radius of 400 μm cladded with PEGDA 700, 40%. (**b**) Photo taken from the lateral side of the PEGDA waveguide. The fabricated waveguides can still guide light even more than 2 months after fabrication. Figure S2: Fabricated straight waveguide (length = 10 cm) with PDMS as the cladding and PEGDA 700, 90 % as the core (radius = 500 μm). Waveguide is extremely flexible as seen in the series of images showing that a significant light output is still present even with increasing waveguide bending. Scale bar is 5 cm. Figure S3: Stability measurements of the fabricated waveguides. (**a**) Output power from the straight waveguide over time for 300 μm, 400 μm, 500 μm waveguide radius. Note that power readings are stable for 30 min. (**b**) Rhodamine fluorescence emission from the microfluidic chip with integrated PEGDA waveguides. The emission intensity is very stable for 25 min. Data presented here are an average of three measurements.
