# Supplementary material for: Fabrication of a Monolithic Lab-on-a-Chip Platform with Integrated Hydrogel Waveguides for Chemical Sensing
Source: Sensors (Basel). 2019 Oct 8;19(19):4333. doi: 10.3390/s19194333 (PMC6806100; doi:10.3390/s19194333)
Supplement: Supplementary file 1 [file sensors-19-04333-s001.zip › sensors-599309-SI.pdf]

Supplementary Material for
"Fabrication of a Monolithic Lab-On-A-Chip Platform with Integrated Hydrogel Waveguides for Chemical Sensing"

Maria Leilani Torres-Mapa[1,5], Manmeet Singh[1,5], Olga Simon[2,5], Jose Louise Mapa[1], Manan Machida[3], Axel Günther[4], Bernhard Roth[4,6], Dag Heinemann[2,5], Mitsuhiro Terakawa[3] and Alexander Heisterkamp[1,2,3,5]
[1] *Institute of Quantum Optics, Gottfried Wilhelm Leibniz University Hannover, Hannover, Germany*
[2] *Industrial and Biomedical Optics Department, Laser Zentrum Hannover e.V., Hannover, Germany*
[3] *School of Integrated Design Engineering, Keio University, Yokohama, Japan*
[4] *Hannover Centre for Optical Technologies, Gottfried Wilhelm Leibniz University Hannover, Hannover, Germany*
[5] *Lower Saxony Centre for Biomedical Engineering, Implant Research and Development (NIFE), Hannover, Germany*
[6] *Cluster of Excellence PhoenixD (Photonics, Optics, and Engineering-Innovation Across Disciplines), Hannover, Germany*
(Dated: October 2, 2019)

TABLE S1: Design dimension of the molds and the corresponding fabricated PEGDA waveguide radii (n=2)

**Design radius (μm)**	**Fabricated waveguide radius (μm)**
300	300± 0
400	525± 35
500	650± 0

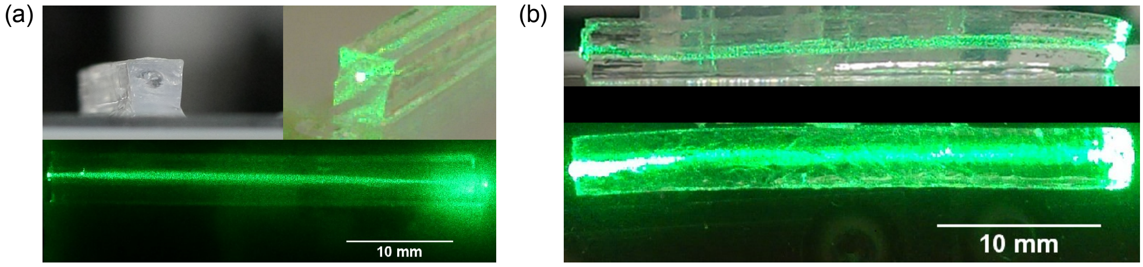

FIG. S1: (**a**) Waveguiding using PEGDA 700, 90% (n= 1.462) as core with a radius of 400 μm cladded with PEGDA 700, 40%. (**b**) Photo taken from the lateral side of the PEGDA waveguide. The fabricated waveguides can still guide light even more than 2 months after fabrication.

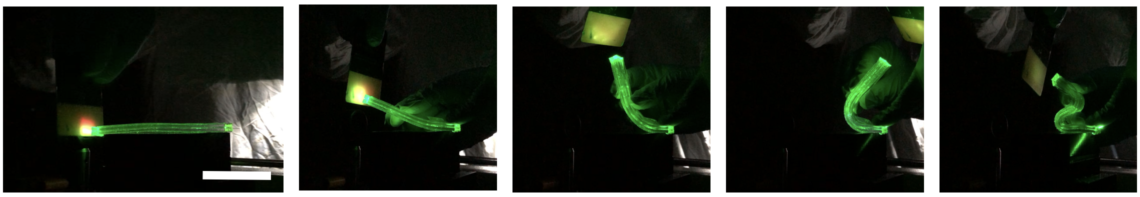

FIG. S2: Fabricated straight waveguide (length = 10 cm) with PDMS as the cladding and PEGDA 700, 90 % as the core (radius = 500 μm). Waveguide is extremely flexible as seen in the series of images showing that a significant light output is still present even with increasing waveguide bending. Scale bar is 5 cm.

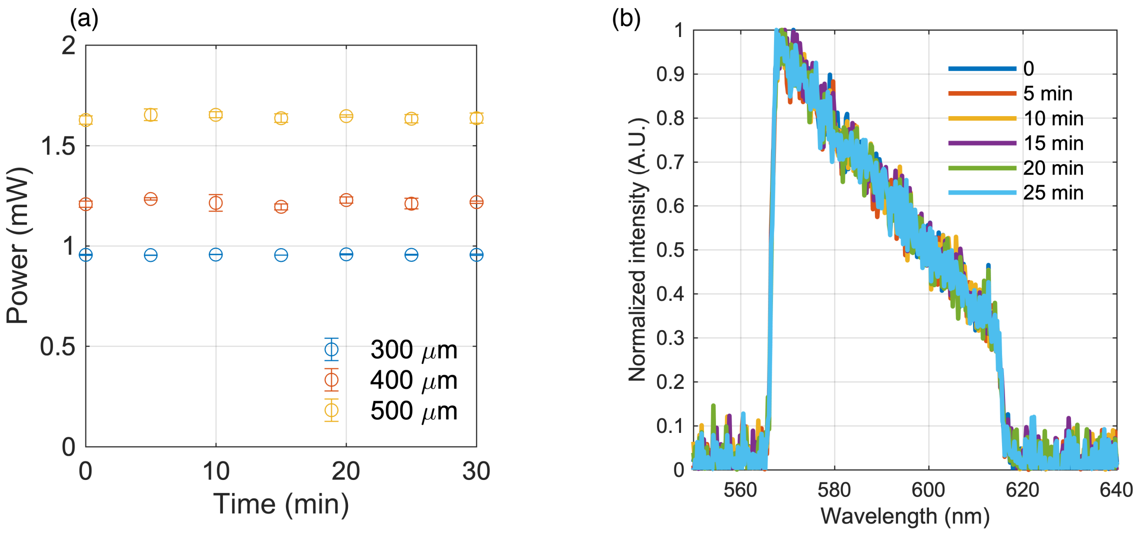

FIG. S3: Stability measurements of the fabricated waveguides. (a) Output power from the straight waveguide over time for 300 μm, 400 μm, 500 μm waveguide radius. Note that power readings are stable for 30 min. (b) Rhodamine fluorescence emission from the microfluidic chip with integrated PEGDA waveguides. The emission intensity is very stable for 25 min. Data presented here are an average of three measurements.
